# Phytochemical investigation of *Ludwigia adscendens* subsp. *diffusa* aerial parts in context of its biological activity

**DOI:** 10.1186/s13065-022-00909-8

**Published:** 2022-12-10

**Authors:** Mostafa H. Baky, Mohamed R. Elgindi, Enas M. Shawky, Haitham A. Ibrahim

**Affiliations:** 1 Department of Pharmacognosy, Faculty of Pharmacy, Egyptian Russian University, Badr City, Cairo, 11829 Egypt; 2 Department of Pharmacognosy, Faculty of Pharmacy, Helwan University, Cairo, Egypt

**Keywords:** *Ludwigia adscendens* subsp. *diffusa*, Onagraceae, Triterpenoids, Flavonoids, Hepatoprotection, Cytotoxicity

## Abstract

*Ludwigia adscendens* subsp. *diffusa* (Onagraceae), an important aquatic herb widely distributed in the Nile River and canals in Egypt. The goal of the current study is to investigate the phytochemical composition of *L. adscendens* aerial parts *n*-butanol and ethyl acetate fractions and screening of its biological activities. Phytochemical investigation of *L. adscendens *resulted in the isolation and purification of eleven compounds belonging to flavonoids, saponins, triterpenoids, and oligosaccharides, of which one compound was identified as new using different spectroscopic techniques. Compound 2 was identified as a new compound namely, 3-*O*-[*β*-D-glucopyranoside (1 → 4) *α*-L-rhamnopyranoside]-23-*O*-feruloyl-hederagenin-28-*O*-[*α*-L-rhamnopyranoside (1 → 2) *β*-D-glucopyranoside], along with other 10 well know compounds. Furthermore, antidiabetic, hepatoprotective and cytotoxic activities of *n*-butanol and ethyl acetate fractions were investigated in vitro, revealing that ethyl acetate fraction was the most active as antidiabetic (IC_50_ = 62.3 µg/mL), hepatoprotective (IC_50_ = 80.75 µg/mL), and cytotoxic against human prostate cancer cell line (IC_50_ = 52.2 µg/mL). Collectively, *L. adscendens *aerial part is rich with a myriad of phytochemicals with potential health benefits.

**Supplementary Information:**

The online version contains supplementary material available at 10.1186/s13065-022-00909-8.

## Introduction

Plants play a pivotal role in drug development process owing to its richness in bioactive phytochemicals with potential health benefits [1, 2]. *Ludwigia* L. (family Onagraceae) is an important pantropic genus widely distributed in South and North America and comprises about 82 species of flowering plants [3]. Several traditional uses including antidiabetic [4], antioxidant, antimicrobial [5], antidiarrheal [6], and anti-inflammatory activity [7] were reported for *Ludwigia* species [8]. Genus *Ludwigia* was reported for its richness in different phytochemicals such as flavonoids, saponins, phenolic compounds, and triterpenes [3, 8]. *L. adscendens *subsp. *diffusa* (Forssk.)P.H.Raven also known as *Ludwigia stolonifera* (Guill. & Perr.)P.H.Raven is a dominant aquatic macrophytes distributed over canals and drains branching from the Nile River in Egypt [9]. *L. adscendens* is important in water remediation by improving water quality by eliminating various toxic pollutants [9, 10]. With the continuous interest in the identification of new phytochemicals with novel structural and biological properties, this study was undertaken for the isolation and purification of different phytochemicals from *L. adscendens* aerial parts by different chromatographic techniques. Eleven compounds were isolated and identified from *L. adscendens* aerial parts *n*-butanol and ethyl acetate fractions. Moreover, the biological activity of both fractions and aerial parts total extracts were investigated showing potential antidiabetic, hepatoprotective, and cytotoxic activities.

## Methods/experimental

### General

NMR analysis was performed using JOEL GX-400 (400 and 100 MHz for 1H and 13C NMR), NMR Laboratory, Faculty of Pharmacy, Cairo University, Cairo, Egypt. All samples were analyzed in DMSO-*d*6 and CD_3_OD-*d*4 solvent. Thin layer chromatography (TLC) was performed on silica gel 60 F254 precoated aluminium sheets (20 × 20, 0.2 mm thikness) and cellulose precoated aluminium sheets (20 × 20, 0.2 mm thikness), (E. Merck, Darmstadt, Germany). Paper Chromatograpy (PC) was performed by Whatmann No.1 (Whatmann Ltd., Maidstone, Kent, England). Aluminium chloride reagent (1% in ethanol) for flavonoids and Ferric chloride reagent (1% in ethanol) were used for spots visualization. Solvent systems S_1_: chloroform: methanol: water ((70:30:5) & (70:30:2) *v/v/v*), S_2_: Chloroform: methanol ((80:20),(70:30)&(50:50) *v*/*v*), S_3_: *n*-butanol: acetic acid: water (BAW) (4:1:5 *v*/*v*/*v*, upper layer) and S_4_: Acetic acid: water (15:85 *v*/*v*). HR-ESI/MS analyses were carried out using a Bruker LC micro-Q-TO-F mass spectrometer, Faculty of Pharmacy, Ain Shams University, Egypt. Additionally, Microplate reader (SunRise, Tecan, USA), 96-well microtiter plates (Greiner, Germany), inverted microscope (Olympus 1 × 70, Tokyo, Japan) and Jouan® centrifuge, 1,000 – 10,000 r.p.m., France for biological studies which were performed at theMycology and Biotechnology Reginal Center, Al-Azhar University.

### Plant material

*Ludwigia adscendens* subsp. *diffusa* (Forssk.) P.H.Raven syn. *Ludwigia stolonifera* (Guill. & Perr.) P.H.Raven (Onagraceae) aerial parts were collected from the Nile River et al.-Qanater Al-Khayriyah, El Qulyoubia governorate, Egypt, at 54VM + 52 in September 2019. The plant was botanically identified by Prof. Dr. Rim Hamdy, Botany Department, Faculty of Science, Cairo University, Egypt. A voucher specimen has been deposited at Pharmacognosy Department, Faculty of Pharmacy, Helwan university No = 31*Lus*1/2022. The air dried coarsely divided aerial parts (1050 g) were macerated in 5 L of 100% methanol with occasional stirring at room temperature and the process was repeated three times (3 × 5 L) till exhaustion. The methanolic extract was concentrated and dried under reduced pressure at 50 ^ο^C to give dry total extract (170 g).

### Chemical reagents

Quercetin, myricetin and monosaccharaides standards were obtained from Sigma/Aldrich, USA. Dimethylsulphoxide (DMSO) was provided from Sigma, St. Louis, CA, USA. Acarbose, silymarin, doxorubicin, PC-3 cells (human prostate cancer cell line) were obtained from The Regional Center for Mycology and Biotechnology, Al-Azhar University, Egypt.

### Extraction and isolation

The dried aerial parts (1050 g) of *L. adscendens* were macerated in 100% methanol (3 × 3L), to yield concentrated methanol extract (170 g). The dried residue (150 g) was reconstituted in 250 ml distilled water and sequentially partitioned and fractionated using different immiscible solvents (petroleum ether, chloroform, ethyl acetate and *n*-butanol solvents). The ethyl acetate fraction (35 g) was subjected to silica G 60 in a glass column (3 × 1.5 mm dimensions) using a step gradient chloroform and methanol mixtures. The fractions were investigated and collected according to their similarities on TLC cellulose plates using S4 solvent system and ammonia spray reagent to afford 10 main collective fractions. Fraction-III (15 g) eluted with 80% CHCl_3_/MeOH, was added on sephadex sub-column and eluted with BAW to afford five main sub-fractions-(1–5) according to TLC cellulose plates. Sub-fraction-3 (3 g) was further purified on sephadex sub-column to afford one pure compound **1** (15 mg). Sub-fraction-4 (6 g) was subjected to more purification on sephadex sub-column to afford two pure compounds **2** (30 mg) and **3** (20 mg). Fraction-IV (12 g) eluted with 70% CHCl_3_/MeOH, was further purified on sephadex column and eluted with BAW to yield five main sub-fractions according to similarity on TLC cellulose plates. Sub fractions-a (4 g) was subjected to more purification on sephadex sub-column to afford two pure compounds 4 (15 mg), and 5 (10 mg). Sub-fraction-b (3 g) was purified using sephadex LH-20 sub-column to afford one pure compound **6** (10 mg). The *n*-butanol fraction (35 g) was mixed with 50 ml MeOH and poured on 250 ml acetone to yield acetone precipitate (25 g) which was subjected to silica gel G 60 in a glass column using step gradient chloroform and menthol mixtures with increasing polarity from 100% CHCl_3_ to 100% MeOH for elution. Fractions were investigated and collected according to TLC silica plates using different solvent system and spray reagents. Fraction-III (9 g) eluted with 80% CHCl_3_/MeOH, was subjected to silica gel sub-column and elution with CHCl_3_/MeOH to afford five main sub-fractions which were collected according to TLC silica plates. Sub-fraction-A (2 g) was purified using a silica gel sub-column to afford one pure compound 7 (10 mg). Sub-fractions-B (5 g) was subjected to more purification by silica gel sub-column to afford two pure compounds 8 (15 mg) and 9 (10 mg). Fraction-IV (11 g) eluted with 70% CHCl_3_/MeOH, was further purified on silica sub-column and elution with CHCl_3_/MeOH to afford four main sub-fractions. Sub-fraction-C (4 g) was then purified by another silica sub-column to yield two pure compounds 10 (15 mg) and 11 (10 mg).

### Antidiabetic activity

The antidiabetic activity of *L. adscendens* aerial parts fractions against acarbose was investigated in vitro using *α*-glucosidase-inhibitory assay as previously mentioned [11]. Briefly, in a 96-well plates a mixture of 50 μL phosphate buffer (100 mM, pH = 6. 8), 10 μL *α*-glucosidase (1U/mL), and 20 μL of each concentration (sample and standard) was pre-incubated for 15 min at 37 ºC. 20 μL of p-Nitrophenol (5 mM) was further added with incubation for 20 min at 37 ºC. 50 μL of Na_2_ CO_3_ (0.1 M) was added and absorbance was measured at 405 nm using Multiplate Reader. The percentage inhibition was calculated using the formula.

$$ {\text{Inhibitory activity }}\left( \% \right) \, = \, \left( {{1 } - {\text{ As}}/{\text{Ac}}} \right) \, \times {1}00 $$

Where, (As) the absorbance in the presence of extract, (Ac) is the absorbance of control.

### Hepatoprotective activity

The hepatoprotective effect of *L. adscendens* aerial part was tested in vitro [12]*.* In brief, 50 μL of MTT (5 mg/mL) was added to each well containing 100 μL rpm hepatocyte suspension. The plates were incubated in the dark at 37 ˚C for an additional 4 h in 5% CO_2_ atmosphere. 150 μL DMSO was added and absorbance was measured at 570 nm with a microplate reader. The results were expressed as percentage of viability calculated as [(ODt/ODc)] × 100%. The 50% Effective concentration (EC_50_) was estimated from graphic plots of the dose–response curve for each conc using Graphpad Prism software and the following equation.

$$ {\text{Hepatoprotective percentage}}\, = \,\% {\text{ Viability of treatment group}}{-}\% {\text{ Viability of negative control}} $$

### Cytotoxicity activity

Cytotoxic activity of *L. adscendens* aerial part fractions were tested *in vitro* using MTT cell viability assay as previously described [13]. Briefly, in each well plate 100 µL of fresh culture RPMI 1640 medium without phenol red then 10 µL of the 12 mM MTT stock solution (5 mg of MTT in 1 mL of PBS) were added to each well. An 85 µL aliquot of the incubated media was replaced by 50 µL of DMSO and incubated at 37 ºC for 10 min. The optical density was measured at 590 nm with the microplate reader to determine the number of viable cells and the percentage of viability was calculated as the percentage of cell survival was calculated as follows:

$$ {\text{Surviving fraction }} = \, \left( {{\text{O}}.{\text{D}}. \, \left( {\text{treated cells}} \right)} \right)/\left( {{\text{O}}.{\text{D}}. \, \left( {\text{control cells}} \right)} \right) \, *{ 1}00 $$

The 50% inhibitory concentration (IC_50_) was estimated from graphic plots of the dose response curve for each concentration using Graphpad Prism software [14].

## Results and discussion

### Characterisation of isolated compounds

**Fig. 1:**
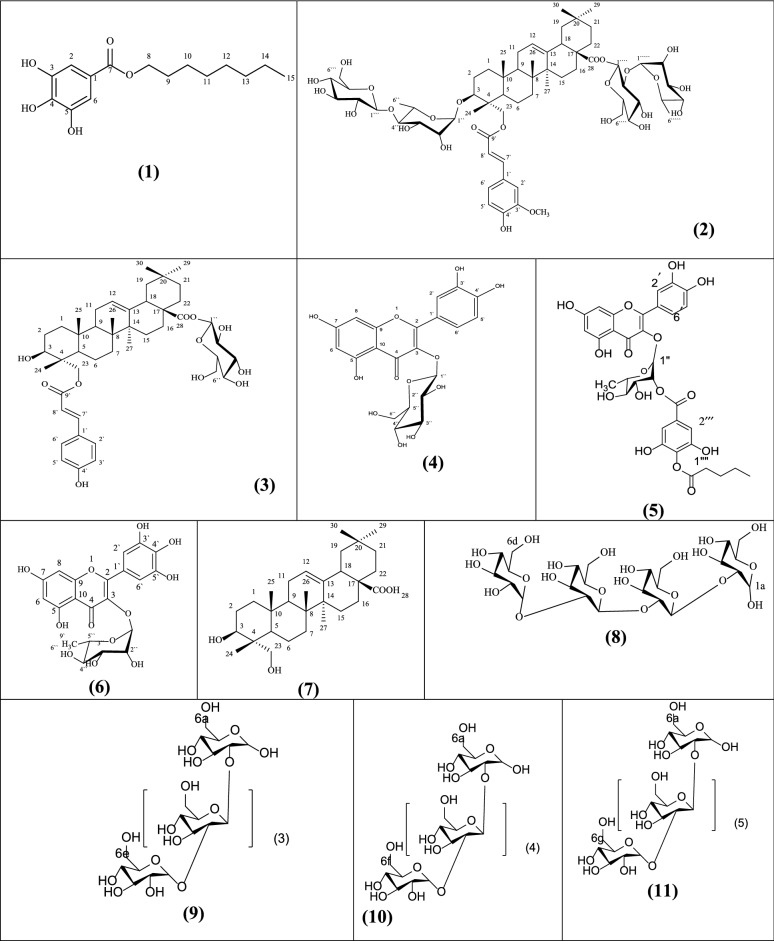
Isolated compounds from *L. adscendens*

Investigation of *L. adscendens* aerial parts ethyl acetate and *n*-butanol fractions resulted in isolation of eleven compounds of which six compounds (1–6)were identified in ethyl acetate versus five compounds (7–11)from *n*-butanol fraction (Fig. 1). Among the isolated compounds, compound 2 was identified as a novel natural compound along with 10 known compounds. Previously isolated compounds included are octyl gallate (1)[15], 23-*O*-Coumaroyl-hederagenin-28-*O*-*β*-D-glucopyranoside (3) [16], quercetin-3-*O*-glucoside (4) [17], quercetin 3-*O*-*α*-L-rhamnoside-2''-(4'''-*O*-n-pentanoyl)-gallate (5) [17], myricetin-3-*O*-*α*-L-rhamnopyranoside (6) [18], hederagenin (7) [19], *α*-D-tetraglucoside (*α*-D-glucopyranosyl-(2a → 1b)-*O*-*α*-D-glucopyranosyl-(2b → 1c)-*O*-*α*-D-glucopyranosyl-(2c → 1d)-*O-α*-D-glucopyranoside) (8) [20], *α*-D-pentaglucoside (*α*-D-glucopyranosyl-(2a → 1b)-*O*-D-glucopyranosyl-(2b → 1c)-*O*-*α*-D-glucopyranosyl-(2c → 1d)-*O*-*α*-D-glucopyranosyl-(2d → 1e)-*O*-*α*-Dglucopyranoside) (9), *α*-D-hexaglucoside (*α*-D-glucopyranosyl-(2a → 1b)-*O*-D-glucopyranosyl-(2b → 1c)-*O*-*α*-D-glucopyranosyl-(2c → 1d)-*O*-*α*-D-glucopyranosyl-(2d → 1e)-*O*-*α*-D-glucopyranosyl-(2e → 1f)-*O*-*α*-D-glucopyranoside) (10), and *α*-D-heptaglucoside (α-D-glucopyranosyl-(2a → 1b)-*O*-D-glucopyranosyl-(2b → 1c)-*O*-*α*-D-glucopyranosyl-(2c → 1d)-*O*-*α*-D-glucopyranosyl-(2d → 1e)-*O*-*α*-D-glucopyranosyl-(2e → 1f)-*O*-*α*-D-glucopyranosyl-(2f → 1 g)-*O*-*α*-D-glucopyranoside) (11).

**Fig. 2:**
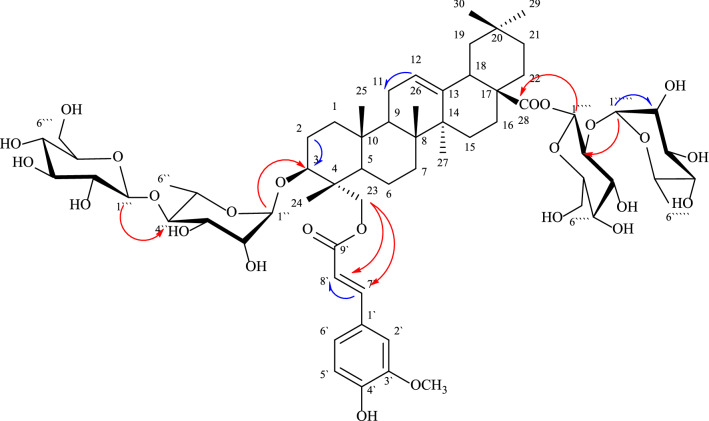
Key 1H − 1H COSY and HMBC correlations of compound 2

Compound 2 was obtained as a white amorphous powder (15 mg), with R*f* = 0.35 on silica gel TLC plate, it gave violet color with 10% conc. H_2_SO_4_ spray reagent. All ^1^H NMR and ^13^C NMR spectroscopic data are summarized in (Table 1). The ^1^H NMR spectrum of compound 2 (Additional file 1: Fig. S1-S2) showed the presence of six singlet signals at *δ* ppm 0.81, 0.89, 0.79, 0.92, 0.85 and 0.87 corresponding to the methyl groups at C-24, C-25, C-26, C-27, C-29 and C-30, respectively. Moreover, the presence of a triplet signal at *δ* ppm 3.44 corresponding to H-3 along with the presence of a broad triplet at δ 5.25 ppm attributed to H-12 of a tri-substituted olefinic bond revealed the presence of a triterpene skeleton [21]. The aromatic region of the ^1^HNMR spectrum of compound 2 displayed the characteristic signals of a feruloyl moiety appearing at δ 7.22 (d), 6.82 (d), 7.4 ppm (dd) for H-2', H-5'and H-6', respectively, alongside the olefinic proton signals appearing as doublet at δ7.61 and 6.28 ppm for H-7'and H-8', respectively. The proton signal of methoxy group appeared as a singlet at δ 3.25 ppm. The ^1^H NMR spectrum of compound 2 showed four signals corresponding to anomeric protons at δ 5.49 (brs), 5.21 (d, *J* = 7.9), 5.42 (d, *J* = 7.9) and 5.37 (s) assigned for H-1'', H-1''', H-1'''', and H-1''''', respectively, revealing the presence of four sugar moieties (two *β*-D-glucoside and two *α*-L-rhamnoside) attached to the aglycone at different positions. Moreover, the characteristic methyl signals of rhamnoside moieties were recorded at δ ppm 1.05 (d, *J* = 6.1) and 1.01 (d, *J* = 6.2). The remaining proton signals of sugars were recorded as typical ranging from δ 3.20 to 4.13 ppm. The ^13^C NMR spectrum of compound **2** (Additional file 1: Fig. S3-S4) displayed the characteristic 30 carbon signals of oleanane-type triterpene moiety matched with hederagenin structure. The ^13^CNMR spectrum of compound 2 showed two key signals for olefinic carbons C-12 at *δ* 121.57 ppm, C-13 at δ 144.98 ppm and a carbonyl carbon C-28 at δ 178 ppm was detected. The downfield shift of C-23 at *δ* 67.57 ppm indicated its substitution with hydroxyl group. Moreover, ^13^C NMR spectrum showed the characteristic 9 carbon signals of ferulic acid at δ ppm 128.47, 108.12, 145.15, 148.37, 115.10, 121.69, 144.92, 115.14 and 167.96 for (C-1' to 9'), respectively alongside carbon signal of methoxy group appearing at δ ppm 55.15. The presence of the feruloyl moiety at C-23 in the aglycone was confirmed by downfield shift of H-23 at δ ppm 3.79 (d) and C-23 carbon at δ ppm 67.75 which coincided with previous literature. ^13^C NMR chemical shifts exhibited four anomeric signals at δ C104.52, 98.45, 93.37 and 102.13 corresponding to C-1'', C-1''', C'''' and C-1''''', respectively indicating the presence of four sugar moieties and another two carbon signals at δ18.62 and 18.62 ppm for C-6'' and C-6''''' respectively, which confirm the presence of two *β*-D-glucoside and two *α*-L-rhamnoside moieties. The bond of sugar moiety at C-28 and C-3 confirmed by spectral data of carbon at δ178.21 and 86.62 ppm and in accordance with literature [22]. The aforementioned ^1^H and ^13^CNMR data were finally confirmed using 2D correlations (Fig. 2) viz, COSY and HMBC (Additional file 1: Fig. S5 and S6). The HMBC spectrum of compound 2 showed a direct ^*2*^* J* correlation between H-23 and H-8' with C-9' confirming feruloyl moiety attachment position to the aglycone part, the direct ^*2*^* J* correlation of H-1'' and H-2'' with C-3 (*δ* 86.62) confirmed the of rhamnose moiety directly at C-3 of the aglycone part, whereas a direct ^*2*^* J* correlation between H-1''' with C-4'' (δ 81.87) confirming the bond of glucose moiety to C-4'' of the rhamnose moiety. On the other hand, direct ^*2*^* J* correlation between H-1''''and H-2''''with C-28 (δ 178.21) confirming bond of glucose moiety to C-28 of the aglycone moiety and a direct ^*2*^* J* correlation between H-1''''' with C-2'''' (*δ* 77.29) confirmed bonding of rhamnose moiety at C-2'''' of the glucose moiety. A direct *J*_H-H_ correlation correlation observed in the COSY spectrum was observed between the anomeric proton of rhamnose and aglycone moiety H-1'' (δ 5.49) with H-3 (*δ* 3.44). Other direct *J*_H-H_ correlation of sugar moieties included between rhamnose moiety anomeric proton H-1'' (*δ* 5.49) with H-2'' (δH 4.11), and the anomeric proton of rhamnose and glucose moiety H-1''''' (δ 5.37) with H-2'''' (δ 3.56). A direct *J*_H-H_ COSY correlations of feruloyl moiety between H-5' (*δ* 6.82) with H-6' (*δ* 7.4) and H-7' (δ 7.61) with H-8' (δ 6.28) of feruloyl moiety confirmed this acyl substituent in compound 2. The LC–MS showed molecular ion peak [M-H]^−^ at *m/z* 1264.6241 (calcd. 1264.4470), consistent with the molecular formula to be C_64_H_96_O_25_^−^ (calcd. C_64_H_97_O_25_). From 1 D, 2 D, and MS data and by comparison with previously reported data [21, 22] compound 2 was asigned as 3-*O*-[*β*-D- glucopyranoside (1 → 4) *α*-L-rhamnopyranoside]-23-*O*-feruloyl-hederagenin-28-*O-*[*α*-L-rhamnopyranoside(1 → 2)*β*-D- glucopyranoside] (Fig. 1)*.*

**Table 1:** NMR Spectroscopic Data for compounds **2**

No.	2	
	**δ**_**H**_ (*J* in Hz) (400 MHz, DMSO-*d*_*6*_)	**δ**_**C**_** ppm** (100 MHz, DMSO-*d*_*6*_)
1	—	39.2
2	—	25.5
3	3.44 m	86.6
4	—	42.7
5	—	48.1
6	1.37 m	18.8
7	1.26 m	31.6
8	—	39.5
9	1.60 m	47.4
10	—	38.7
11	1.82 m	22.3
12	5.25 (brt, 4.8)	121.5
13	—	144.9
14	—	41.9
15	1.44 m	28.7
16	2.1 m	22.6
17	—	47.2
18	2.46 m	41.6
19	1.30 m	45.8
20	—	30.3
21	1.17 m	33.7
22	1.75 m	30.2
23	3.79 m	67.7
24	0.81 s	12.8
25	0.89 s	16.2
26	0.79 s	18.6
27	0.92 s	26.7
28	—	178.2
29	0.85 s	34.3
30	0.87 s	23.5
1'	—	128.4
2'	7.22 (d, 7.9)	108.1
3'	—	145.1
4'	—	148.3
5'	6.82 (d, 7.9)	115.1
6'	7.4 (dd, 7.9, 4)	121.6
7'	7.61 (d, 11.9)	144.9
8'	6.28 (d, 7.9)	115.1
9'	—	167.9
OCH_3_	3.25 s	55.1
3-*O*-rh		
1''	5.49 (br.s)	104.5
2''	4.11 (m)	73.9
3''	3.8 (m)	70.6
4''	3.42 (m)	81.8
5''	4.13 (m)	69.7
6''	1.05 (d, 6.1)	18.6
glu(terminal)		
1'''	5.21 (d, 7.9)	98.4
2'''	3.44 m	76.8
3'''	3.50 m	76.7
4'''	3.46 m	71.7
5'''	3.30 m	75.5
6'''	3.65 d	61.3
	3.80 d	
28-*O*-glu		
1''''	5.42 (d, 7.9)	93.3
2''''	3.56 m	77.2
3''''	3.26 m	76.5
4''''	3.20 m	73.7
5''''	3.21 m	76.1
6''''	4.10 m	61.3
	4.13 m	
Rha(terminal)		
1'''''	5.37 (s)	102.1
2'''''	4.09 m	71.8
3'''''	3.65 m	70.5
4'''''	3.21 m	70.7
5'''''	4.23 m	65.2
6'''''	1.01 (d, 6.2)	18.6

### Antidiabetic activity

The in vitro antidiabetic potential of *L. adscendens* aerial parts total extract, ethyl acetate fraction, and *n*-butanol fraction at different concentrations was assessed using *α*-glucosidase inhibition assay compared to acarbose as standard antidiabetic drug, Additional file 1: Fig. S8a. The calculated IC_50_ for acarbose and the different fractions of *L. adscendens* are listed in Table 2 and Additional file 1: Fig. S8b. The results revealed that, compared to *n*-butanol fraction and total extract, *L. adscendens* ethyl acetate fraction showed the strongest α-glucosidase enzymes inhibition effect with IC_50_ value of 62.30 µg/mL compared to that of acarbose (30.57 µg/mL). The activity of ethyl acetate fraction was due to it its richness in different phytochemicals such as flavonoids and triterpenoids [17]. Results were in accordance with that reported by Marzouk et al., 2007 for a potential hypoglycemic effect of *L. adscendens* subsp. *diffusa* (*Jussiaea repens*) aerial parts ethyl acetate extract in alloxan-induced diabetic rat model [17].

**Table 2:** Calculated IC_50_ (µg/mL) of acarbose and different fractions of *Ludwigia adscendens* subsp. *diffusa* aerial parts for antidiabetic activity, Calculated EC_50_ for silymarin and different fractions of *L. adscendens* for hepatoprotective activity and Calculated IC_50_ (µg/mL) of different fractions of *L. adscendens* against PC-3 cell line

Fractions	*α*-glucosidase inhibitory IC_50_(µg/mL)	Hepatoprotective activity EC_50_(µg/mL)	Cytotoxcity activity IC_50_(µg/mL)
Ethyl acetate fraction	62.30	80.75 ± 21.83	52.2 ± 2.9
*n*-butanol fraction	> 1000	97.96 ± 54.61	77.1 ± 5.2
Aerial parts total extract	133.60	143.24 ± 61.74	182 ± 8.1
Acarbose	30.57		
silymarin		39.64 ± 2.61	
Doxorubicin			34.91 ± 5.21

### Hepatoprotective activity

Hepatoprotective activity of *L. adscendens* aerial parts total extract, ethyl acetate fraction, and *n*-butanol fraction at different concentrtions were likewise assessed on hepatocyte cell damage by MTT-assay compared to silymarin as standard hepatoprotective drug, Additional file 1: Fig. S9a. The calculated EC_50_ for silymarin and different fractions of *L. adscendens* are labelled in Table 2 and Additional file 1: Fig. S9b. The results showed that different concentrations of ethyl acetate and *n*-butanol fractions have moderate hepatoprotective effect against MTT hepatocyte damage with EC_50_ value of 80.75, 97.96 µg/mL, respectively, compared to EC_50_ value of 39.64 µg/mL of standard hepatoprotective drug. Such hepatoprotective potential of *L. adscendens* fractions is owing to its richness in antioxidant constituents such as polyphenols and flavonoids which protect hepatocyte from damage [3].

### Cytotoxicity activity

The cytotoxic activity against of *L. adscendens* aerial parts total extract, ethyl acetate fraction, and *n*-butanol fraction at different concentrtions was determined in vitro against PC-3 cell line (prostate carcinoma cells). The obtained results of the different fractions were expressed as a mean value of cell growth inhibition (Additional file 1: Fig. S10a). The calculated IC_50_ for the different fractions are summarized in Table 2 and Additional file 1: Fig. S10b. Results showed that among tested extract and fractions, ethyl acetate fraction showed the highest cytotoxic activity against PC-3 cell line, with IC_50_ value of 52.2 µg/mL. The cytotoxic effect of *L. adscendens* was consistent with previous reports on Onagraceae species [3] such as *Oenothera paradoxa* revealing for a potential effect to prevent human prostate cancer cells proliferation [23].

## Conclusion

*Ludwigia adscendens* subsp. *diffusa* is an important herbaceous aquatic plant in the Nile Delta region in Egypt. This study aimed towards the isolation and structural elucidation of phytochemicals from *L. adscendens* aerial parts ethyl acetate and *n*-butanol fractions. Eleven compounds were identified from ethyl acetate and *n*-butanol fractions of which one was identified as a novel compound assigned as 3-*O*-[*β*-D-glucopyranoside(1 → 4)*α*-L-rhamnopyranoside]-23-*O*-feruloyl-hederagenin-28-*O-*[*α*-L-rhamnopyranoside(1 → 2)*β*-D- glucopyranoside] (2). Biological investigation of *L. adscendens* aerial parts total extract, ethyl acetate fraction, and *n*-butanol fraction revealed that ethyl acetate fraction was most active as antidiabetic, hepatoprotective and cytotoxic. Finally, further studies are recommended to isolate phytochemicals from other classes from *L. adscendens* and in vivo antidiabetic and hepatoprotective studies are recommended to prove for its efficacy as revealed from this in vitro based assay to be conclusive.

## Supplementary Information

**Additional file1: Figure S1.** Magnification of 1H NMR spectrum of compound 2. **Figure S2.** Magnification of 1H NMR spectrum of compound 2. **Figure S3.** Magnification of ^13^C NMR spectrum of compound 2. **Figure S4.** Magnification of ^13^C NMR spectrum of compound 2. **Figure S5.** HMBC spectrum of compound 2. **Figure S6.** COSY spectrum of compound 2. **Figure S7.** MS spectrum of compound 2. **Figure S8.** (a) Hepatoprotective activity of different concentrations of silymarin and different fractions of the *L. adscendens *aerial parts. **(b)** Calculated EC50 (µg/ml) for silymarin and different fractions of *L. adscendens *aerial parts. **Figure S9.** (a) Hepatoprotective activity of different concentrations of silymarin and different fractions of the *L. adscendens *aerial parts. (b) Calculated EC50 (µg/ml) for silymarin and different fractions of *L. adscendens *aerial parts. **Figure S10.** (a) Cytotoxic activity of different concentrations of different fractions of *L. adscendens *against PC-3 cell line.** (b) **Calculated IC_50_ (µg/ml) of different fractions of *L. adscendens *against PC-3 cell line. **Table S1.** NMR Spectroscopic Data for Compound 1. **Table S2.** NMR Spectroscopic Data for Compounds 2, 3 and 7. **Table S3. **NMR Spectroscopic Data for Compounds 4, 5 and 6. **Table S4.** NMR Spectroscopic Data for Compounds 8, 9, 10 and 11.

## Data Availability

All data generated or analysed during this study are included in this published article [and its supplementary information files].
