# Supplementary material for: Phytochemical investigation of Ludwigia adscendens subsp. diffusa aerial parts in context of its biological activity
Source: BMC Chem. 2022 Dec 10;16(1):112. doi: 10.1186/s13065-022-00909-8 (PMC9737734; doi:10.1186/s13065-022-00909-8)
Supplement: Supplementary file 1 — Additional file1: Figure S1. Magnification of 1H NMR spectrum of compound 2. Figure S2. Magnification of 1H NMR spectrum of compound 2. Figure S3. Magnification of 13C NMR spectrum of compound 2. Figure S4. Magnification of 13C NMR spectrum of compound 2. Figure S5. HMBC spectrum of compound 2. Figure S6. COSY spectrum of compound 2. Figure S7. MS spectrum of compound 2. Figure S8. (a) Hepatoprotective activity of different concentrations of silymarin and different fractions of the L. adscendens aerial parts. (b) Calculated EC50 (µg/ml) for silymarin and different fractions of L. adscendens aerial parts. Figure S9. (a) Hepatoprotective activity of different concentrations of silymarin and different fractions of the L. adscendens aerial parts. (b) Calculated EC50 (µg/ml) for silymarin and different fractions of L. adscendens aerial parts. Figure S10. (a) Cytotoxic activity of different concentrations of different fractions of L. adscendens against PC-3 cell line. (b) Calculated IC50 (µg/ml) of different fractions of L. adscendens against PC-3 cell line. Table S1. NMR Spectroscopic Data for Compound 1. Table S2. NMR Spectroscopic Data for Compounds 2, 3 and 7. Table S3. NMR Spectroscopic Data for Compounds 4, 5 and 6. Table S4. NMR Spectroscopic Data for Compounds 8, 9, 10 and 11. [file 13065_2022_909_MOESM1_ESM.docx]

**Supplementary Materials**

**Phytochemical investigation of *Ludwigia adscendens* subsp. *diffusa* aerial parts in context of its biological activity**

**Mostafa H. Baky^a^***, **Mohamed R. Elgindi^b^**, **Enas M. Shawky^a^**, **Haitham A. Ibrahim^b^**

^a^ *Department of Pharmacognosy, Faculty of pharmacy, Egyptian Russian University. Badr city, 11829, Cairo, Egypt.*

^b^ *Department of Pharmacognosy, Faculty of pharmacy, Helwan University, Cairo, Egypt.*

**Abstract**

*Ludwigia adscendens* subsp. *diffusa* (Onagraceae), an important aquatic herb widely distributed in the Nile River and canals in Egypt. The goal of the current study is to investigate the phytochemical composition of *L. adscendens* aerial parts *n*-butanol and ethyl acetate fractions and screening of its biological activities. Phytochemical investigation of *L. adscendens* resulted in the isolation and purification of eleven compounds belonging to flavonoids, saponins, triterpenoids, and oligosaccharides, of which one compound was identified as new using different spectroscopic techniques. Compound 2 was identified as a new compound namely, 3-*O*-[*β*-D-glucopyranoside (1→4)*α*-L-rhamnopyranoside]-23-*O*-feruloyl-hederagenin-28-*O*-[*α*-L-rhamnopyranoside(1→2)*β*-D-glucopyranoside], along with other 10 well know compounds. Furthermore, antidiabetic, hepatoprotective and cytotoxic activities of *n*-butanol and ethyl acetate fractions were investigated *in vitro*, revealing that ethyl acetate fraction was the most active as antidiabetic (IC_50_=62.3µg/mL), hepatoprotective (IC_50_ =80.75µg/mL), and cytotoxic against human prostate cancer cell line (IC_50_=52.2µg/mL). Collectively, *L. adscendens* aerial part is rich with a myriad of phytochemicals with potential health benefits.

**Key words**

*Ludwigia adscendens* subsp. *diffusa*; Onagraceae; triterpenoids; flavonoids; hepatoprotection; cytotoxicity

| **Supplementary materials Contents:** | |
| --- | --- |
| **Figure S1** | Magnification of 1H NMR spectrum of compound 2 |
| **Figure S2** | Magnification of 1H NMR spectrum of compound 2 |
| **Figure S3** | Magnification of ^13^C NMR spectrum of compound 2 |
| **Figure S4** | Magnification of ^13^C NMR spectrum of compound 2 |
| **Figure S5** | HMBC spectrum of compound 2 |
| **Figure S6** | COSY spectrum of compound 2 |
| **Figure S7** | MS spectrum of compound 2 |
| **Fig. S8** | **(a)** Antidiabetic activity of different concentrations of acarbose and different fractions of the *L. adscendens* aerial parts. **(b)** Calculated IC_50_ (µg/ml) of Acarbose and different fractions of *L. adscendens* aerial parts. |
| **Fig. S9** | **(a)** Hepatoprotective activity of different concentrations of silymarin and different fractions of the *L. adscendens* aerial parts. **(b)** Calculated EC50 (µg/ml) for silymarin and different fractions of *L. adscendens* aerial parts. |
| **Fig. S10** | **(a)** Cytotoxic activity of different concentrations of different fractions of *L. adscendens* against PC-3 cell line. **(b)** Calculated IC_50_ (µg/ml) of different fractions of *L. adscendens* against PC-3 cell line. |
| **Table S1** | NMR Spectroscopic Data for Compound 1 |
| **Table S2** | NMR Spectroscopic Data for Compounds 2, 3 and 7 |
| **Table S3** | NMR Spectroscopic Data for Compounds 4, 5 and 6 |
| **Table S4** | NMR Spectroscopic Data for Compounds 8, 9, 10 and 11 |

**Figure S1. Magnification of ^1^HNMR spectrum of compound 2**

**Figure S2. Magnification of ^1^HNMR spectrum of compound 2**

**Figure S3. Magnification of ^13^CNMR spectrum of compound 2**

**Figure S4. Magnification of ^13^CNMR spectrum of compound 2**

**Figure S5. HMBC spectrum of compound 2**

**Figure S6. COSY spectrum of compound 2**

**
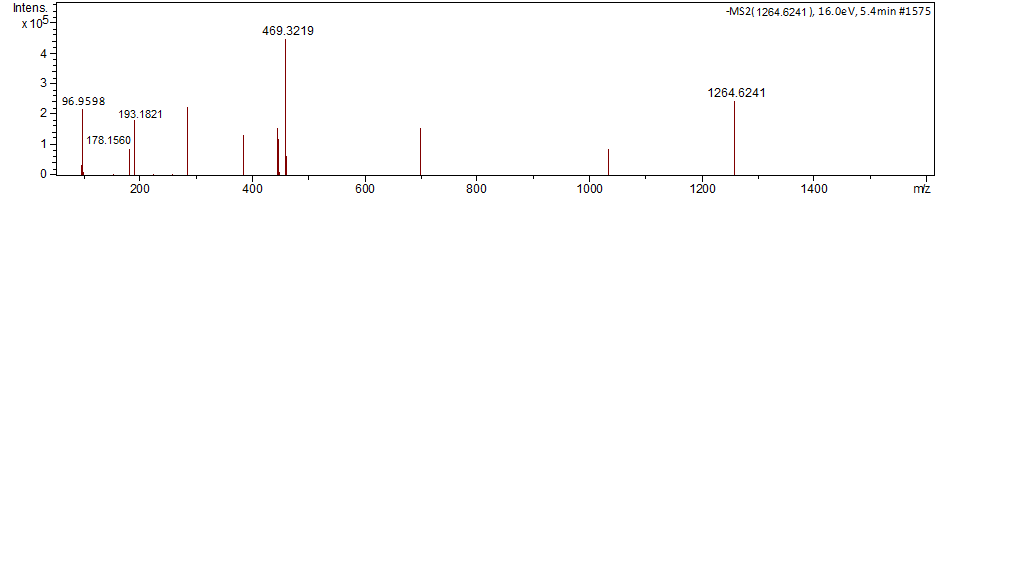
**

**Figure S7. MS spectrum of compound 2**


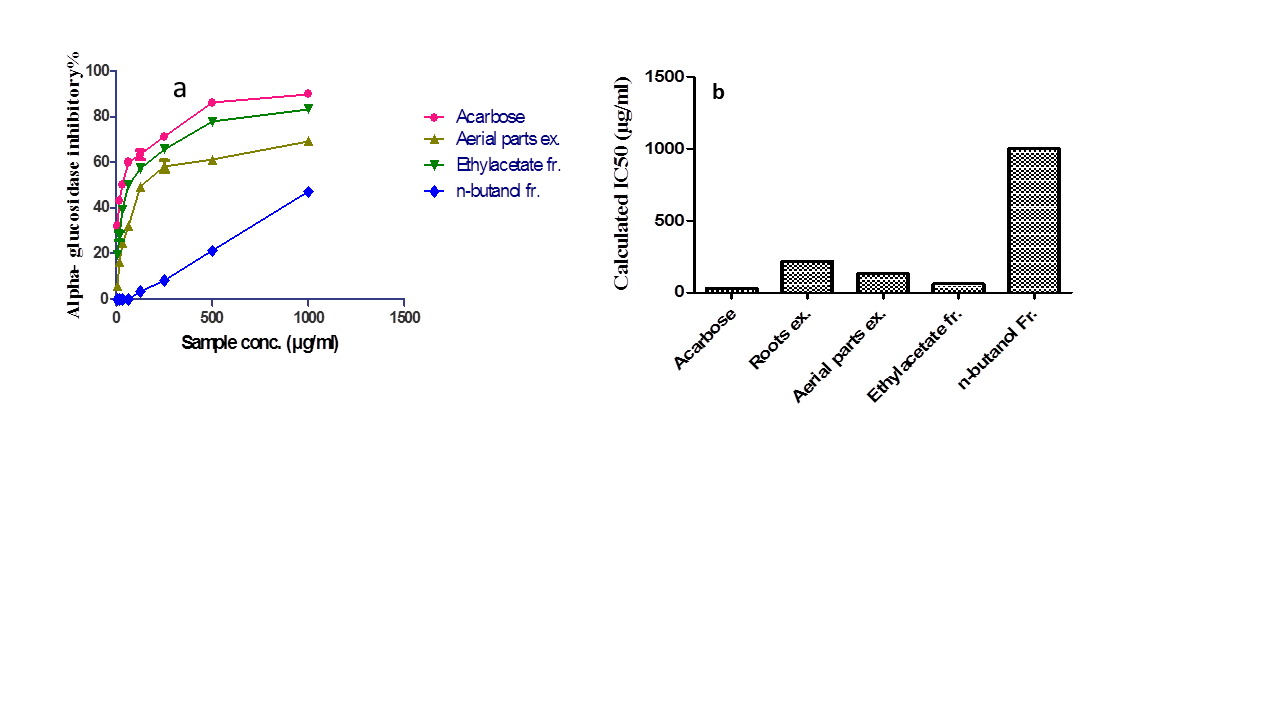


**Fig. S8 (a)** Antidiabetic activity of different concentrations of acarbose and different fractions of the *L. adscendens* aerial parts. **(b)** Calculated IC_50_ (µg/ml) of Acarbose and different fractions of *L. adscendens* aerial parts.


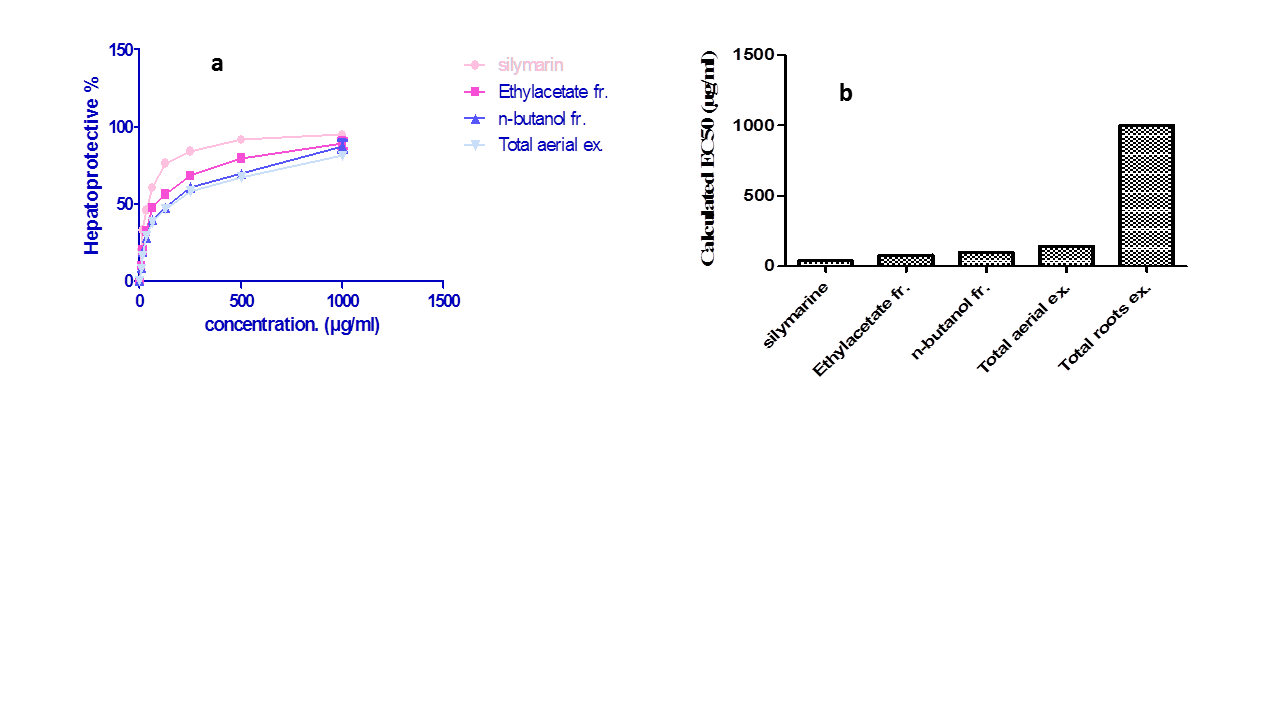


**Fig. S9 (a)** Hepatoprotective activity of different concentrations of silymarin and different fractions of the *L. adscendens* aerial parts. **(b)** Calculated EC50 (µg/ml) for silymarin and different fractions of *L. adscendens* aerial parts.


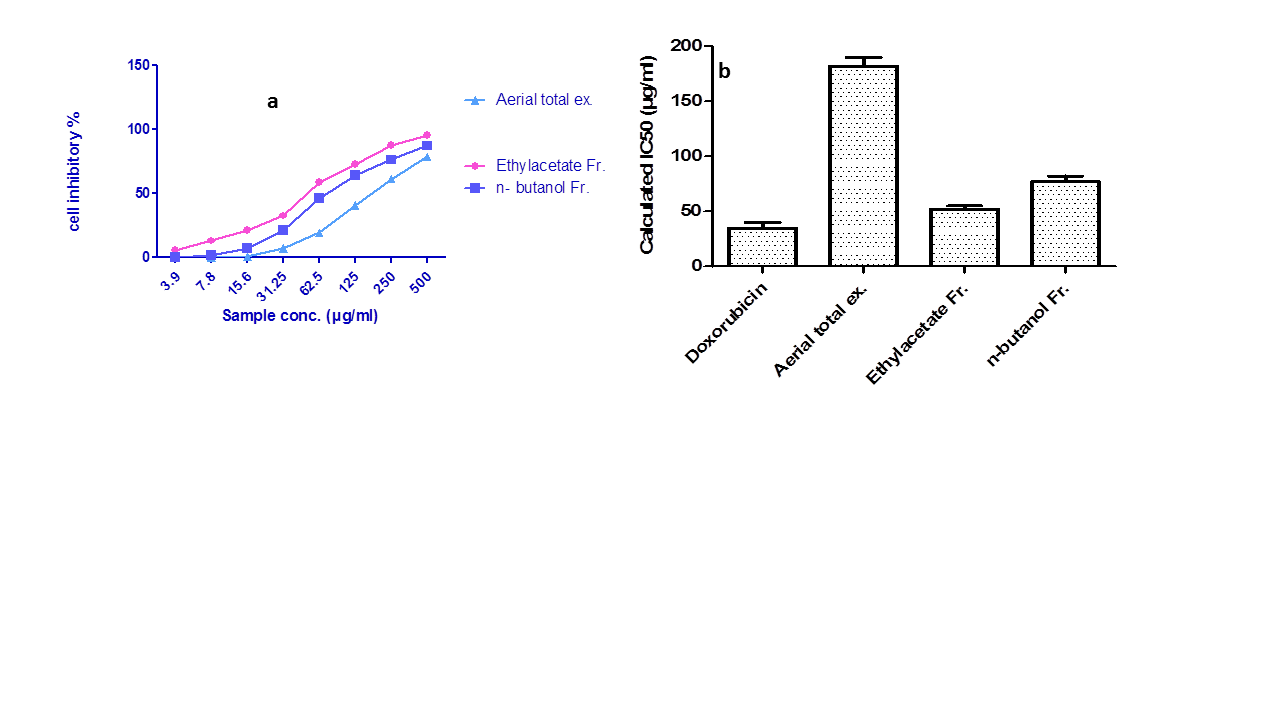


**Fig. S10 (a)** Cytotoxic activity of different concentrations of different fractions of *L. adscendens* against PC-3 cell line. **(b)** Calculated IC_50_ (µg/ml) of different fractions of *L. adscendens* against PC-3 cell line.

| **Table S1. NMR Spectroscopic Data for Compound 1 in DMSO-*d_6_*** | | |
| --- | --- | --- |
| **No.** | **δ_H_** (*J* in Hz) | **δ_C_ ppm** |
| OH proton | 2.45 s |  |
|  | --- | 120.7 |
|  | 6.96 s | 108.9 |
|  | --- | 144.9 |
|  | --- | 138.1 |
|  | --- | 144.9 |
|  | 6.96 s | 108.9 |
|  | --- | 169.1 |
|  | 4.18 t (7, 6.3) | 65.3 |
|  | 1.64 m | 38.7 |
|  | 1.35 m | 30.3 |
|  | 1.30 m | 28.7 |
|  | 1.24 m | 23.5 |
|  | 1.24 m | 22.6 |
|  | 1.24 m | 18.8 |
|  | 0.87 t (8, 6.3) | 12.6 |

| **Table S2. NMR Spectroscopic Data for Compounds 3 and 7 in**  CDCl_3_ | | | | | |
| --- | --- | --- | --- | --- | --- |
| No. | 3 | | | 7 | |
|  | **δ_H_** (*J* in Hz) | | **δ_C_ ppm** | **δ_H_** (*J* in Hz) | **δ_C_ ppm** |
| 1 | 0.99 m | | 38.1 | 1.50 m | 37.7 |
| 2 | **---** | | 24.5 | **---** | 27.8 |
| 3 | 3.43 m | | 73.2 | 3.55 m | 71.6 |
| 4 | **---** | | 42.8 | **---** | 43.5 |
| 5 | 1.10 m | | 48.2 | 1.31 m | 48.2 |
| 6 | **---** | | 19.1 | 1.42 m | 19.8 |
| 7 | 1.32 m | | 31.6 | **---** | 35.1 |
| 8 | --- | | 38.7 | **---** | 41 |
| 9 | --- | | 47.8 | **---** | 48.0 |
| 10 | --- | | 35.3 | **---** | 35.9 |
| 11 | 1.96 m | | 23.8 | **---** | 23.4 |
| 12 | 5.24 br t | | 121.5 | 5.24 br t | 122.8 |
| 13 | **---** | | 145.1 | **---** | 144 |
| 14 | **---** | | 42 | **---** | 41.5 |
| 15 | 1.04 m | | 28.0 | 2.15 m | 28.9 |
| 16 | 1.93 m | | 22.8 | 2.23 m | 23.0 |
| 17 | **---** | | 46.4 | **---** | 45.5 |
| 18 | 2.45 m | | 41.4 | 3.49 m | 42.1 |
| 19 | **---** | | 44.9 | **---** | 46.2 |
| 20 | **---** | | 29.1 | **---** | 31.6 |
| 21 | **---** | | 35.8 | 1.13 m | 35.9 |
| 22 | **---** | | 30.7 | 2.09 m | 33.3 |
| 23 | 3.79 m | | 68.6 | 4.05 m | 65.5 |
| 24 | 0.82 s | | 13.5 | 0.85 s | 13.0 |
| 25 | 0.87 s | | 14.5 | 0.78 s | 16.0 |
| 26 | 0.78 s | | 17.7 | 0.82 s | 17.5 |
| 27 | 0.92 s | | 25.4 | 0.87 s | 26.8 |
| 28 | **---** | | 178.2 | **---** | 183 |
| 29 | 0.86s | | 33.4 | 0.77 s | 33.8 |
| 30 | 0.85 s | | 21.3 | 0.80 s | 22.3 |
| 1' | **---** | | 124.9 |  |  |
| 2' | 7.49 (d, 8) | | 134.8 |  |  |
| 3' | 6.99 (d, 8) | | 116.3 |  |  |
| 4' | --- | | 157. |  |  |
| 5' | 6.99 (d, 8) | | 116.3 |  |  |
| 6' | 7.49 (d, 8) | | 134.8 |  |  |
| 7' | 7.26 (d,12) | | 145.0 |  |  |
| 8' | 6.75 (d, 8) | | 114.9 |  |  |
| 9' | **---** | | 165.7 |  |  |
| OCH_3_ |  | |  |  |  |
| 3-O-rh |  | |  |  |  |
| 1'' |  | |  |  |  |
| 2'' |  | |  |  |  |
| 3'' |  | |  |  |  |
| 4'' |  | |  |  |  |
| 5'' |  | |  |  |  |
| 6'' |  | |  |  |  |
| glu(terminal) |  | |  |  |  |
| 1''' |  | |  |  |  |
| 2''' |  | |  |  |  |
| 3''' |  | |  |  |  |
| 4''' |  | |  |  |  |
| 5''' |  | |  |  |  |
| 6''' |  | |  |  |  |
|  |  | |  |  |  |
| 28-O-glu |  | |  |  |  |
| 1'''' | 1´´ | 5.08(d,7.8) | 102.5 |  |  |
| 2'''' | 2´´ | 3.36 m | 70.7 |  |  |
| 3'''' | 3´´ | 3.46 m | 71.7 |  |  |
| 4'''' | 4´´ | 3.54 m | 73.6 |  |  |
| 5'''' | 5´´ | 3.63 m | 75.7 |  |  |
| 6'''' | 6´´ | 3.75 m | 60.5 |  |  |
|  |  | |  |  |  |
| Rha(terminal) |  | |  |  |  |
| 1''''' |  | |  |  |  |
| 2''''' |  | |  |  |  |
| 3''''' |  | |  |  |  |
| 4''''' |  | |  |  |  |
| 5''''' |  | |  |  |  |
| 6''''' |  | |  |  |  |

| **Table S3. NMR Spectroscopic Data for Compounds 4, 5 and 6** in DMSO-*d_6_* | | | | | | |
| --- | --- | --- | --- | --- | --- | --- |
| No. | 4 | | 5 | | 6 | |
|  | **δ_H_** (*J* in Hz) | **δ_C_ ppm** | **δ_H_** (*J* in Hz) | **δ_C_ ppm** | **δ_H_** (*J* in Hz) | **δ_C_ ppm** |
| 2 | --- | 157.6 | --- | 161.5 | --- | 156.9 |
| 3 | --- | 134.4 | --- | 134.8 | --- | 134.5 |
| 4 | --- | 178.1 | --- | 178.4 | --- | 177.9 |
| 5 | --- | 161.6 | --- | 164.2 | --- | 161.6 |
| 6 | 6.20 (d,4) | 100.3 | 6.16 s | 98.5 | 6.14 (d, 2) | 99.6 |
| 7 | --- | 165.1 | --- | 164.3 | --- | 166.6 |
| 8 | 6.40 (d,4) | --- | 6.32 s | 93.4 | 6.32 (d, 2) | 94.2 |
| 9 | --- | 156.4 | --- | 157.8 | --- | 157 |
| 10 | --- | 106.2 | --- | 104.5 | --- | 104.4 |
| 1´ | --- | 120.6 | --- | 121.6 | --- | 119.9 |
| 2´ | 7.24 ( d, 2) | 114.4 | 7.3 (d,4) | 115.7 | 6.89 s | 108.3 |
| 3´ | --- | 146.0 | --- | 144.8 | --- | 146.3 |
| 4´ | --- | 147.7 | --- | 148.3 | --- | 137.2 |
| 5´ | 6.68 (d, 8) | 115.7 | 6.93 (d,8) | 115.0 | --- | 146.3 |
| 6´ | 7.18(dd,2,8.4) | 121.3 | 7.5 (dd,8.5,2) | 122.0 | 6.89 s | 108.3 |
| 1´´ | *β*-D-glucopyranoside | | *α*-L-rhamnoside | | *α*-L-rhamnoside | |
|  | 5.21 d (7.8) | 104.2 | 5.47 s | 102. | 5.21 br s | 102.3 |
| 2´´ | 3.77 m | 81.8 | 3.94 m | 80.5 | 3.25 m | 71.7 |
| 3´´ | 3.64 m | 74.3 | 3.83 m | 70.6 | 3.18 m | 70.9 |
| 4´´ | 3.81 m | 72.0 | 3.44 m | 71.9 | 3.11 m | 73.3 |
| 5´´ | 3.60 m | 78.7 | 3.39 m | 61.4 | 3.16 m | 69..2 |
| 6´´ | 3.67 m | 63.5 | 0.98 (d, 5.8) | 18.6 | 0.86(d, 8) | 18.0 |
| 1´´´ |  |  | --- | 121.6 |  |  |
| 2´´´/6´´´ |  |  | 7.3 s | 108.1 |  |  |
| 3´´´/5´´´ |  |  | --- | 145.0 |  |  |
| 4´´´ |  |  | --- | 133.4 |  |  |
| 7´´´ |  |  | --- | 174.3 |  |  |
| 1´´´´ |  |  | --- | 178.1 |  |  |
| 2´´´´ |  |  | 2.36 t | 34.3 |  |  |
| 3´´´´ |  |  | 1.5 tt of pentet-like | 20.0 |  |  |
| 4´´´´ |  |  | 1.35 tq of sixtet-like | 16.3 |  |  |
| 5´´´´ |  |  | 0.91 t | 12.9 |  |  |

| **Table S4. NMR Spectroscopic Data for Compounds 8, 9, 10 and 11** in CD3OD-*d_4_* | | | | | | | | |
| --- | --- | --- | --- | --- | --- | --- | --- | --- |
| **NO.** | **8** | | **9** | | **10** | | **11** | |
|  | **δ_H_** (*J* in Hz) | **δ_C_ ppm** | **δ_H_** (*J* in Hz) | **δ_C_ ppm** | **δ_H_**(*J* in Hz) | **δ_C_ ppm** | **δ_H_** (*J* in Hz) | **δ_C_ ppm** |
| 1a | 4.85 (d, 4) | 104.6 | 4.85( d, 4) | 104.5 | 4.94 (d, 4) | 104.6 | 4.87 (d, 3.2) | 104.6 |
| 2a | 3.85 m | 83.3 | 3.81 m | 83.3 | 3.82 m | 83.2 | 3.85 m | 83.3 |
| 3a | 3.71 m | 71.9 | 3.44 m | 72.8 | 3.24 m | 72.7 | 3.42 m | 72.9 |
| 4a | 3.53 m | 70.3 | 3.42 m | 70.3 | **---** | 69.5 | 3.4 m | 70.3 |
| 5a | 3.82 m | 76.3 | 3.75 m | 77.1 | 3.44 m | 76.1 | 3.73 m | 77.1 |
| 6a | 3.36 d(d, 8) | 63.4 | 3.12 (d, 4) | 61.5 | 3.13 ( d, 8) | 63.4 | 3.58 (d, 4) | 64.8 |
| 1b | 4.67 (d, 8) | 102.4 | 4.71 (d, 6.28) | 102.4 | 4.81 (d,4) | 102.3 | 4.84 (d, 4.5) | 102.4 |
| 2b | 3.79 m | 82.3 | 3.79 m | 82.3 | 3.78 m | 82.2 | 3.82 m | 82.3 |
| 3b | 3.69 m | 70.9 | 3.73 m | 72.3 | 3.20 m | 70.9 | 3.41 m | 72.8 |
| 4b | 3.73m | 68.2 | **---** | 70.7 | 3.06 m | 70.2 | 3.47 m | 69.6 |
| 5b | 3.75 m | 76.1 | 3.68 m | 76.2 | 3.41 m | 75.9 | 3.70 m | 76.3 |
| 6b | 3.30 (d, 5.16) | 63.3 | 3.10 (d, 8) | 61.6 | 3.22 (d, 8) | 63.3 | 3.52 (d, 8) | 64.1 |
| 1c | 4.54 (d, 4) | 98.4 | 4.49 (d, 4.16) | 98.4 | 4.74 (d 4) | 98.5 | 4.80(d, 5.24) | 98.5 |
| 2c | 3.72 m | 81.3 | 3.83 m | 81.3 | 3.72 m | 81.4 | 3.81 m | 82.3 |
| 3c | 3.41 m | 69.6 | 3.30 m | 75.2 | **---** | 68.1 | 3.39 m | 73.5 |
| 4c | 3.40 m | 64.8 | **---** | 69.6 | 3.01 m | 64.7 | 3.29 m | 68.2 |
| 5c | 3.49 m | 75.7 | 3.58 m | 76.1 | 3.14 m | 75.6 | 3.60 m | 76.1 |
| 6c | 3.28(d,5.16) | 61.5 | 2.91 (d, 8) | 63.3 | 3.02 (d,8) | 63.4 | 3.44 (d, 4) | 63.7 |
| 1d | 4.35 (d, 4) | 97.7 | 4.35 (d, 4) | 97.3 | 4.55 (d,4) | 97.8 | 4.73 (d, 6) | 97.7 |
| 2d | 3.76 m | 72.9 | 3.57 m | 81.3 | 3.69 m | 77.1 | 3.79 m | 81.3 |
| 3d | 3.65 m | 68.3 | 3.29 m | 71.0 | 3.10 m | 70.6 | 3.35 m | 71.0 |
| 4d | 3.38 m | 64.1 | **---** | 68.2 | **---** | 63.5 | **---** | 68.2 |
| 5d | 3.52 m | 75.9 | 3.55 m | 75.7 | 3.38 m | 75.2 | 3.55 m | 75.7 |
| 6d | 3.25(d,6.16) | 59.2 | 2.89 (d, 8) | 63.5 | 2.95 (d, 8) | 61.5 | 3.42 (d, 6) | 63.6 |
| 1e |  |  | 4.27 (d, 6) | 92.6 | 4.45 (d,4) | 97.2 | 4.69 (d, 6) | 97.5 |
| 2e |  |  | 3.5 m | 76.2 | 3.75 m | 76.3 | 3.77 m | 77.1 |
| 3e |  |  | 3.28 m | 73.5 | **---** | 66.9 | 3.31 m | 70.5 |
| 4e |  |  | 3.06 m | 68.4 | **---** | 63.6 | 3.27 m | 67. |
| 5e |  |  | 3.49 m | 77.1 | 3.29 m | 72.8 | 3.53m | 75.2 |
| 6e |  |  | 2.87 (d,8) | 64.1 | 2.89 m | 60.0 | 3.31(d, 7) | 63.6 |
| 1f |  |  |  |  | 4.30 (d,4) | 92.5 | 4.36(d, 4.4) | 92.6 |
| 2f |  |  |  |  | 3.48 m | 72.3 | 3.75 m | 75.7 |
| 3f |  |  |  |  | --- | 71.8 | 3.30 m | 70.7 |
| 4f |  |  |  |  | --- | 64.0 | **---** | 70.3 |
| 5f |  |  |  |  | 3.34 m | 73.4 | 3.50 m | 76.1 |
| 6f |  |  |  |  | 2.86 (d,8) | 59.2 | 3.27(d, 7.5) | 61.6 |
| 1g |  |  |  |  |  |  | 4.26 (d, 6) | 92.3 |
| 2g |  |  |  |  |  |  | 3.71 m | 72.3 |
| 3g |  |  |  |  |  |  | **---** | 69.6 |
| 4g |  |  |  |  |  |  | **---** | 64.8 |
| 5g |  |  |  |  |  |  | 3.44 m | 71.9 |
| 6g |  |  |  |  |  |  | 3.25 ( d, 8) | 61.5 |
